# Predicting Chinese older adults' intention to live in nursing homes using an integrated model of the basic psychological needs theory and the theory of planned behavior

**DOI:** 10.3389/fpubh.2022.947946

**Published:** 2022-10-19

**Authors:** Ming Lei, Jirawan Deeprasert, Rita Yi Man Li, Natchuda Wijitjamree

**Affiliations:** ^1^Rattanakosin International College of Creative Entrepreneurship, Rajamangala University of Technology Rattanakosin, Nakhon Pathom, Thailand; ^2^Department of Economics and Finance, Faculty of Commerce, Hong Kong Shue Yan University, Hong Kong, Hong Kong SAR, China; ^3^Faculty of Humanities, Kasetsart University, Bangkok, Thailand

**Keywords:** Chinese older adults, nursing home, basic psychological needs satisfaction, the theory of planned behavior, intention

## Abstract

The growing number of aging populations has become a major problem worldwide. Nursing homes play an essential role in the later life of older adults. Previous research indicated potential associations between external factors and older adults' intention to live in nursing homes. However, intrinsic motivation has yet to be fully understood. This article addresses an academic void that integrated the Basic Psychological Needs Theory (BPNT) and the Theory of Planned Behavior (TPB) to explore older adults' intentions to live in nursing homes. More specifically, it tested the effects of autonomy, competence, and relatedness needs satisfaction as defined in the BPNT on attitudes, subjective norms, perceived behavioral control, and live-in intentions toward nursing homes in the TPB. An online survey provided quantitative data from 425 aging people. The results indicated that the higher the satisfaction of the basic psychological needs (i.e., autonomy needs, competence needs, and relatedness needs) of the older adults, the lower their intention to live in nursing homes. Furthermore, social pressure partially mediates this relationship. That is, the higher the satisfaction of the basic psychological needs of older adults, the lower the pressure from society. Thus, they should be admitted to the nursing home, and the lower their intention to live in nursing homes. The results contribute to a better understanding of the deep psychological motivation of the older adults' intention to live in nursing homes and support further development of the BPNT-TPB model in older adults' health research.

## Introduction

Addressing an aging population has long become a question of great interest in various fields. China is one of the countries with the fastest aging population (1). While many older adults live separately from their adult children, leading to more and more “empty nest” families (2), older adults' frailty raises the need for nursing homes. In the last few decades, there has been a surge of interest in older adults' intention to live in nursing homes (3). Previous studies on the intention to live in nursing homes have typically concentrated on demographic factors (4–7) and children's financial and emotional support (4, 8, 9). Nevertheless, the rapid changes in China's demographic, social and economic conditions weaken the traditional home care system (3).

It is observed that the integration model of self-determination theory (SDT) and planned behavior theory have yielded promising results in different studies (10–13). Nevertheless, no study has investigated the self-determination theory's role in the older adults' intention to live in nursing homes, not to mention the use of the integration model. The basic psychological needs theory is one of the six mini theories of self-determination theory (14). Given the above, this paper has two aims. First, it investigates the effect of three psychological needs satisfaction on the older adults' intention to live in nursing homes. Second, test the Chinese older adults' intention to live in the nursing home using the integration model.

## Literature review and hypothesis development

### Nursing homes

Sanford et al. (15) have reached an international consensus on the definition of the “nursing home” and what type of services the “nursing home” provides. Therefore, the nursing home in this context refers to a facility with a domestic design that provides 24-h functional support and care to older adults who need help with their daily activities, who often have complex health needs and increased vulnerability. The older adults in nursing homes are self-care, semi-self-care, and non-self-care elders. Most nursing homes mainly accept residents who need long-term care. Most of the employees in nursing homes are trained nurses.

### Basic psychological needs theory

According to Ryan et al. (16), everyone must meet three needs to protect their mental health and the best human functions. The three most basic needs are “autonomy,” “competence” and “relatedness.” Autonomy needs satisfaction refers to the feeling that the individual is free to initiate, maintain, and terminate the target behavior (17). Competence needs satisfaction refers to the perception that one can influence the environment in an ideal way and complete a task within the scope of his ability (17); relatedness needs satisfaction refers to an individual's feeling that they can maintain a good relationship with significant others: care for each other and support each other (18). These needs are cross-personal and cross-cultural and apply to every aspect of life (19). Meeting these needs is essential; they guide people's behavior and are a potential behavioral incentive (17, 20).

### Theory of planned behavior

Ajzen (21) proposed that attitude, subjective norm, and perceived behavioral control together form behavioral intentions. In TPB theory, the intention is defined as an “indication of how hard people are willing to try, of how much effort they are planning to exert, performing the behavior” (21). Attitude refers to an individual's positive or negative evaluation of a specific intention and behavior. Perceived behavioral control is used to describe whether the behavior is complex and whether it is under their control (21). Subjective norms can be defined as the social pressure perceived by significant others or reference groups (22).

### BPNT-TPB framework

A common criticism of predicting human behavior using TPB or basic psychological needs theory basic psychological needs theory (BPNT) alone concerns a lack of motivation. The theory of planned behavior is believed to overlook the origin of social cognitive beliefs; self-determination theory fails to explain the contingency of situations nor the process of transforming motivational orientation into behavioral intentions and behaviors (23, 24).

**Figure 1:**
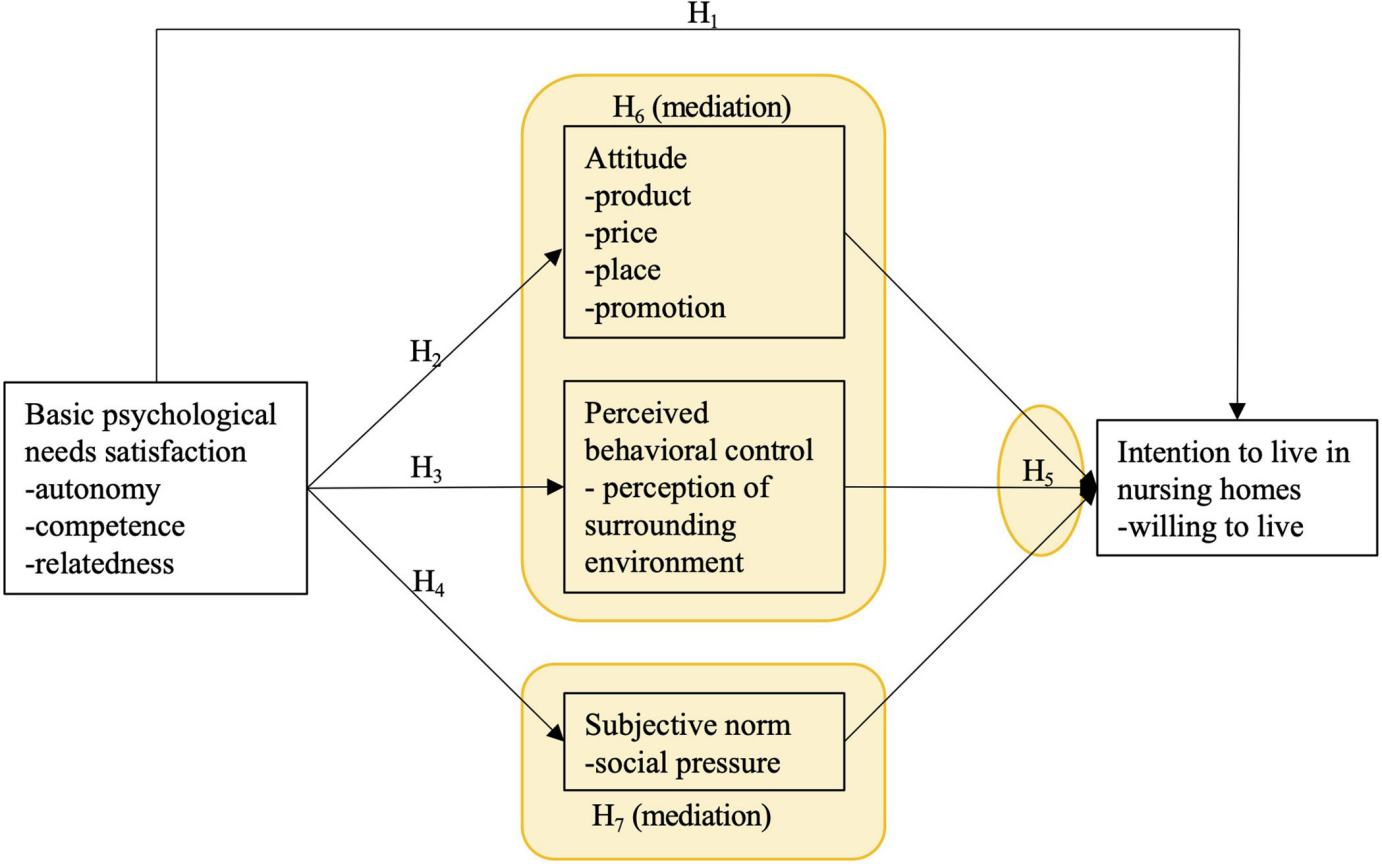
Framework model.

Some scholars tend to integrate self-determination theory with the theory of planned behavior to address these shortcomings. Drawing on 45 tests of SDT-TPB structures, Hagger and Chatzisarantis (12) used meta-analysis to examine the motivation sequence. The results indicated that the proximal predictors of TPB partially mediated the significant effect of SDT on intention. The basic psychological needs theory is the most widely used SDT's six mini-theories (14). Consequently, this study assumed that the BPNT-TPB model is appropriate for studying older adults' intentions to live in nursing homes. This study classifies essential psychological needs satisfaction into three dimensions: autonomy needs satisfaction, competence needs satisfaction and relatedness satisfaction (16), and explores the more complicated relationship between BPNT and TPB variables. The framework model is as shown in Figure 1.

### Development of hypotheses

#### Relationship between basic psychological needs satisfaction and intention

Al-Jubari (25) suggested that autonomic motivation occurs when an individual's behavior satisfies basic psychological needs, namely autonomy, competence, and relatedness. Hagger (26) pinpointed those essential psychological needs satisfaction, directly and indirectly, affected intention. Besides, according to Harris et al. (27), the SDT suggested that people who have been adequately satisfied with psychological needs were more likely to engage in actions to sustain their sense of autonomy, competence, and relatedness needs. Therefore, older adults who meet basic psychological needs in their daily lives may continue their original lifestyle to meet their needs of autonomy, competence, and relatedness. They are expected to be less willing to live in nursing homes. So, this research proposes hypothesis 1:

H1: Basic psychological needs satisfaction negatively influences the intention to live in nursing homes.

#### Relationship between basic psychological needs satisfaction and TPB variables

Individuals align their cognitions and emotions with overall motivation, resulting in self-motivated behaviors to meet the basic psychological needs of autonomy, competence, and relatedness (28). Deci et al. (29) pointed out that the satisfaction of people's needs for autonomy, competence, and relatedness facilitates internalizing their values and supervision processes, which was verified in another study (30). González-Cutre (31) concluded that TPB lacks the origin of the belief, and an individual's pursuit of basic psychological need satisfaction provides the reasons for attitude, intention and behavioral engagement. These research results agreed that the basic psychological need satisfaction predicts proximal factors of TPB: attitudes, subjective norms and perceived behavioral control.

Previous research has concluded that the satisfaction of three basic psychological needs positively affects individual exercise participation in sports (32). According to Baard et al. (33), people with higher satisfaction with their needs usually have a positive attitude toward the target behavior and find it easier to exhibit positive behavior. Similarly, Baard et al. (33) suggested that the higher people's satisfaction with the three needs, the higher their self-esteem and welfare. Al-Jubari (25) researched college students' entrepreneurial intentions and found that satisfaction with basic psychological needs positively affected entrepreneurial attitude, perceived behavioral control, and the subjective norm.

Therefore, this study speculate that for the Chinese older adults, the higher the basic psychological needs satisfaction, the more positive their attitude toward continuing their original life, but the more negative their attitude toward nursing homes. At the same time, there will be less pressure on significant others around them to put them in nursing homes, and the subjective norm will be lower. On the contrary, the higher basic psychological needs satisfaction of the older adults are associated with a higher the perceived behavioral control. This research proposes hypotheses 2–4:

H2: Basic psychological needs satisfaction negatively influences attitude.

H3: Basic psychological needs satisfaction negatively influences subjective norms.

H4: Basic psychological needs satisfaction positively influences perceived behavioral control.

#### Relationship among TPB variables

In a seminal paper, Ajzen (21) provides the earliest description of TPB that behavioral intention is a function of attitude, subjective norm, and perceived behavioral control. In a recent nursing home study, Huang's (34) research show that attitude and perceived behavioral control directly affect the intention to move into a nursing home. Subjective norms have the most negligible effect on intention but strongly affect intention through attitude. The mediation effect shows that their opinions and social perceptions will affect attitudes and thus affect behavioral intentions of moving into a nursing home. At the same time, perceived behavioral control also strongly influences the intention of older adults to move into nursing homes. No matter how positive their attitude toward moving into senior housing is nor how significant others support their decision, their intention to move in will decrease as long as there are potential restrictions. Therefore, this study proposes hypotheses 5–7:

H5: Attitude, subjective norms, and perceived behavioral control positively influence the intention to live in nursing homes.

#### Mediation effect on TPB variables

Many scholars have integrated SDT and TPB to explain the processes behind the behavior. Hagger's (26) motivational sequence research found that basic psychological needs satisfaction affects intentions *via* attitudes and perceived behavioral control. At the same time, the subjective norm's effect is insignificant. This view is supported by Barkoukis (35), who found that the proximal predictors of TPB: attitude, subjective norm, and perceived behavioral control mediate the effect of basic psychological need satisfaction on the intention to exercise leisure time. Similarly, Al-Jubari (25) underlines that attitude and perceived behavioral control completely mediate the effect of satisfying the basic psychological needs of college students' entrepreneurial intention. Therefore, we hypothesized that the basic psychological needs satisfaction indirectly impacts intention *via* attitude, subjective norms and perceived behavioral control. Therefore, this research proposes hypotheses 8–10:

H6: Attitude and perceived behavioral control mediate the effect of basic psychological needs satisfaction on the intention to live in nursing homes.

H7: Subjective norms play a mediating role in the effect of basic psychological needs satisfaction on the intention to live in nursing homes.

## Methods

### Population and sampling technique

This study employed a cross-sectional survey method and distributed electronic questionnaires from January to February 2022. The target population was “the older adults over 60 years old, living in Henan, China, sober-minded, able to use a smartphone.” According to the sampling formula (36), a minimum of 387 samples should be drawn to ensure accuracy. Our study collected a total of 425 valid questionnaires.

### Respondents characteristics

A total of 425 respondents completed the questionnaire, 117 were male and 248 were female. 64.5% of respondents were aged 60–70 years old. 30.4% of respondents had income between 3,001 and 4,000 yuan. 28.0% of respondents were educated in high school. 46.4% of respondents had only one child, and 48.7% of respondents' physical condition was completely healthy. Table 1 provides an overview of the respondent's gender, age, income, education, number of children, and physical condition.

**Table 1:** Descriptive statistics of respondents.

**Attribute**	**Category**	**Number**	**Percentage**
Gender	Male	177	41.6
	Female	248	58.4
Age	60–70	274	64.5
	71–80	125	29.4
	81–90	20	4.7
	91 and above	6	1.4
Income	0–1000 yuan	52	12.2
	1001–2000 yuan	33	7.8
	2001–3000 yuan	72	16.9
	3001–4000 yuan	129	30.4
	4001–5000 yuan	108	25.4
	5001 and above	31	7.3
Education	uneducated	33	7.8
	primary school	61	14.4
	junior high school	90	21.2
	high school	119	28.0
	college	94	22.1
	Undergraduate	20	4.7
	Master degree and above	8	1.9
Number of children	0	7	1.6
	1	197	46.4
	2	149	35.1
	3	56	13.2
	4 and above	16	3.8
Physical condition	completely healthy	207	48.7
	chronically ill but able to take care of themselves	197	46.4
	chronically ill and unable to take care of themselves	21	4.9
	Bedridden and unable to take care of them self	0	0

### Research instrument and statistical analysis

This study used a 6-point scale to let respondents choose (37). The research instrument was scaled that has been validated. Basic psychological needs satisfaction was measured using the psychological satisfaction portion of Chen et al.'s (38) scale, attitude referenced by Purnomo et al.'s (39) scale (attitude to 4Ps: product, price, place, and promotion), subjective norm and perceived behavioral control used Nsoh's (40) scale, intention adopts Xie et al.'s (41) scale. The software used are SPSS and AMOS. The key statistical analysis used is descriptive, reliability, validity, correlation, regression, mediating effect analysis and structural equation modeling.

## Results

### Reliability analysis

Reliability analysis uses Cronbach's alpha reliability coefficient to check the consistency of questionnaire research variables on each measurement item. It can be seen from Table 2 that Cronbach's alpha coefficient of each variable is greater than the standard of 0.7 (0.862–0.921), indicating that the variable has good internal consistency reliability. The “Corrected item-total Correlation” number is greater than the standard of 0.5 (0.645–0.832), indicating that the measurement items meet the research requirements. “Cronbach's Alpha if Item Deleted” shows that deleting any item will not cause Cronbach's alpha value to increase, indicating that the variable has good reliability (42).

**Table 2:** Reliability analysis.

**Variable**	**Item**	**Corrected item-total correlation**	**Cronbach's Alpha if item deleted**	**Cronbach's Alpha**
BPNS-AU	AU1	0.770	0.798	0.862
	AU2	0.745	0.809	
	AU3	0.664	0.842	
	AU4	0.662	0.843	
BPNS-CO	CO1	0.775	0.880	0.904
	CO2	0.792	0.874	
	CO3	0.811	0.867	
	CO4	0.764	0.885	
BPNS-RE	RE1	0.789	0.848	0.891
	RE2	0.764	0.858	
	RE3	0.757	0.861	
	RE4	0.729	0.871	
AT-PD	AT1	0.723	0.899	0.911
	AT2	0.721	0.899	
	AT3	0.703	0.901	
	AT4	0.712	0.900	
	AT5	0.746	0.896	
	AT6	0.775	0.893	
	AT7	0.737	0.897	
AT-PR	AT8	0.749	0.870	0.895
	AT9	0.763	0.867	
	AT10	0.742	0.872	
	AT11	0.750	0.870	
	AT12	0.705	0.880	
AT-PL	AT13	0.645	0.881	0.887
	AT14	0.732	0.862	
	AT15	0.750	0.858	
	AT16	0.776	0.852	
	AT17	0.743	0.861	
AT-PM	AT18	0.692	0.914	0.921
	AT19	0.703	0.913	
	AT20	0.724	0.912	
	AT21	0.729	0.912	
	AT22	0.680	0.914	
	AT23	0.700	0.913	
	AT24	0.700	0.913	
	AT25	0.686	0.914	
	AT26	0.699	0.913	
	AT27	0.707	0.913	
SN	SN1	0.816	0.882	0.910
	SN2	0.760	0.893	
	SN3	0.776	0.890	
	SN4	0.741	0.898	
	SN5	0.775	0.890	
PBC	PBC1	0.801	0.820	0.887
	PBC2	0.773	0.846	
	PBC3	0.767	0.852	
IN	IN1	0.822	0.874	0.910
	IN2	0.816	0.876	
	IN3	0.832	0.864	

BPNS-AU, Basic psychological needs satisfaction-autonomy satisfaction; BPNS-CO, Basic psychological needs satisfaction-competence satisfaction; BPNS-RE, Basic psychological needs satisfaction-relatedness satisfaction; AT-PD, attitude to product; AT-PR, attitude to price; AT-PL, attitude to place; AT-PM, attitude to promotion; SN, subjective norm; PBC, perceived behavioral control; IN, intention to live in nursing homes.

### Convergent and discriminant validity

Table 3 shows that the standardized factor loadings of each item are greater than 0.6 (0.687–0.894), the combined reliability (CR) is more significant than 0.7 (0.853–0.921) and the average variation extraction (AVE) is more significant than 0.5 (0.540–0.776), indicating that each variable has good convergent validity (43).

**Table 3:** Convergent validity analysis of scales.

**Variables**	**Items**	**Factor loading**	**CR**	**AVE**
BPNS	AU	0.830	0.879	0.671
	CO	0.773		
	RE	0.713		
BPNS-AU	AU1	0.871	0.863	0.613
	AU2	0.822		
	AU3	0.715		
	AU4	0.712		
BPNS-CO	CO1	0.828	0.905	0.705
	CO2	0.842		
	CO3	0.868		
	CO4	0.819		
BPNS-RE	RE1	0.857	0.891	0.672
	RE2	0.822		
	RE3	0.817		
	RE4	0.782		
AT	ATPD	0.692	0.853	0.592
	ATPR	0.792		
	ATPL	0.813		
	ATPM	0.775		
AT-PD	AT1	0.756	0.911	0.595
	AT2	0.763		
	AT3	0.738		
	AT4	0.745		
	AT5	0.792		
	AT6	0.825		
	AT7	0.777		
AT-PR	AT8	0.799	0.895	0.630
	AT9	0.833		
	AT10	0.801		
	AT11	0.794		
	AT12	0.738		
AT-PL	AT13	0.687	0.889	0.617
	AT14	0.776		
	AT15	0.807		
	AT16	0.831		
	AT17	0.817		
AT-PM	AT18	0.731	0.921	0.540
	AT19	0.734		
	AT20	0.754		
	AT21	0.765		
	AT22	0.707		
	AT23	0.732		
	AT24	0.733		
	AT25	0.717		
	AT26	0.734		
	AT27	0.738		
PBC	PBC1	0.885	0.888	0.726
	PBC2	0.840		
	PBC3	0.830		
IN	In1	0.879	0.912	0.776
	In2	0.869		
	In3	0.894		

BPNS, basic psychological needs satisfaction; BPNS-AU, Basic psychological needs satisfaction-autonomy satisfaction, BPNS-CO, Basic psychological needs satisfaction-competence satisfaction; BPNS-RE, Basic psychological needs satisfaction-relatedness satisfaction; AT, attitude to 4P; AT-PD, attitude to product; AT-PR, attitude to price; AT-PL, attitude to place; AT-PM, attitude to promotion; SN, subjective norm; PBC, perceived behavioral control; IN, intention to live in nursing homes.

CFA can also be used to analyse the discriminant validity of the scale. Table 4 presents the results of the discriminant validity analysis of the scale. The diagonal line in the table is the AVE square root value, and the rest are the correlation coefficients. The AVE root of each factor is greater than the standardized correlation coefficient outside the diagonal, so the scale has good discriminant validity. Therefore, based on the above analysis, the scales of this study have good validity (43).

**Table 4:** Discrimination validity: Pearson correlation and AVE square root.

	**BPNS**	**ATP**	**SN**	**PBC**	**IN**
BPNS	**0.819**				
AT	−0.144^**^	**0.769**			
SN	−0.223^**^	0.230^**^	**0.820**		
PBC	−0.107^*^	0.197^**^	0.211^**^	**0.852**	
IN	−0.431^**^	0.521^**^	0.653^**^	0.466^**^	**0.881**

Bolded values are AVE root values; BPNS, basic psychological needs satisfaction; AT, attitude to 4P; SN, subjective norm; PBC, perceived behavioral control; IN, intention to live in nursing homes.

* *P* < 0.05,

** *P* < 0.01.

### Hypothesis testing, model fit and path analysis

**Figure 2:**
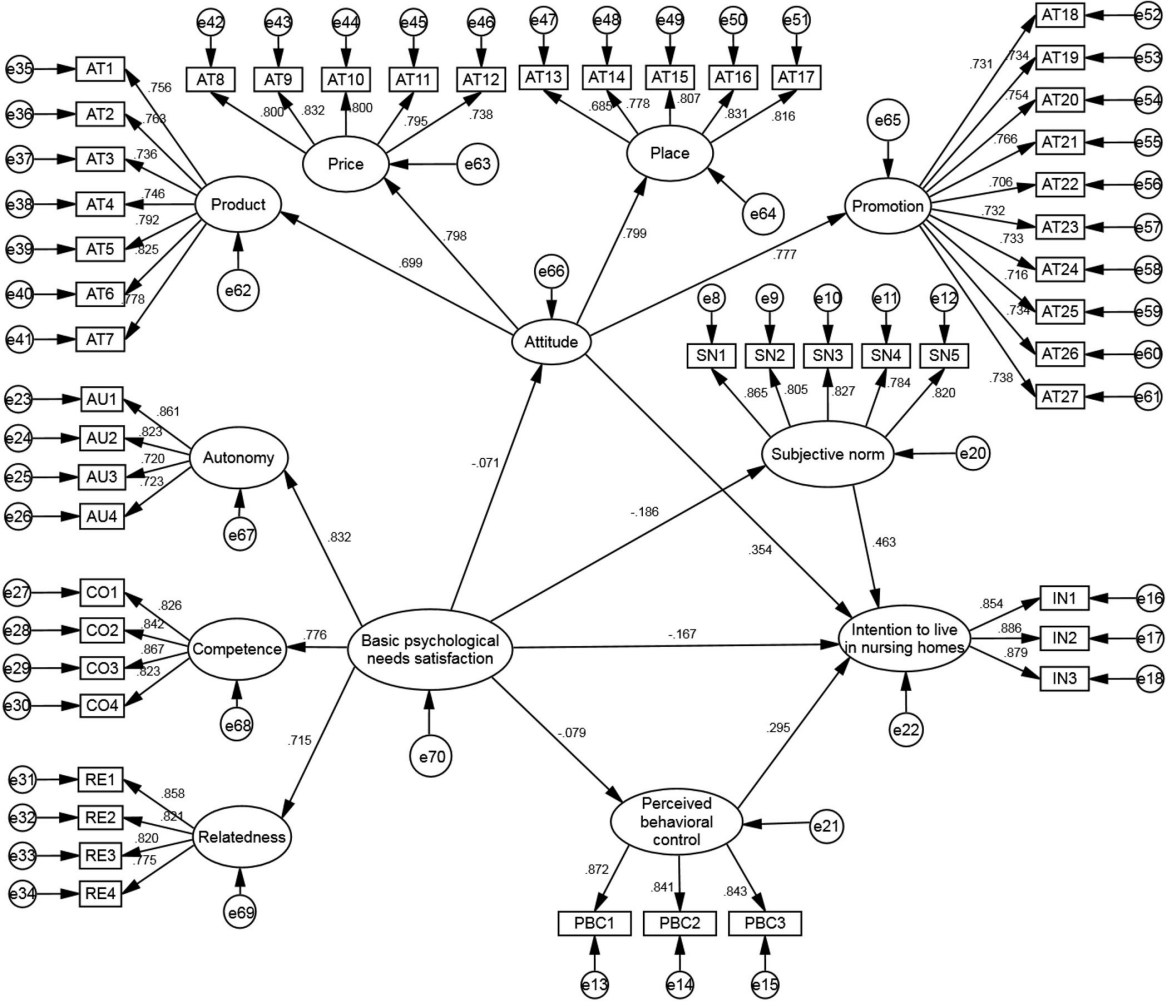
Structural equation model.

This study used a structural equation model, as shown in Figure 2, where the hypotheses were tested concerning the model fit, path coefficient, and mediation test.

The results of the model fit analysis are presented in Table 5. Table 5 showed that CMIN/DF = 1.249, GFI = 0.887, AGFI = 0.873, NFI = 0.903, IFI = 0.979, TLI = 0.978, CFI = 0.979, and RMSEA = 0.024, which indicated that the fitness of the measurement model was acceptable.

**Table 5:** Model fit index.

**Fit index**	**Standard**	**Value**	**Fitting situation**
CMIN	—	1441.366	—
DF	—	1157	—
CMIN/DF	< 3	1.249	Good
RMR	< 0.08	0.058	Good
GFI	>0.9	0.887	Acceptable
AGFI	>0.9	0.873	Acceptable
NFI	>0.9	0.903	Good
IFI	>0.9	0.979	Good
TLI	>0.9	0.978	Good
CFI	>0.9	0.979	Good
RMSEA	< 0.08	0.024	Good

Table 6 summarizes the results obtained from the preliminary analysis. Basic psychological needs satisfaction significantly negatively affects intention (β = −0.167, *p* < 0.001), so H1 is supported. Basic psychological needs satisfaction has no significant effect on attitude (β = −0.071, *p* > 0.05), so H2 is not supported. Basic psychological needs satisfaction significantly negatively affects subjective norm (β = −0.186, *p* < 0.01), so H3 is supported. Basic psychological needs satisfaction has no significant effect on perceived behavioral control (β = −0.079, *p* > 0.05), so H4 is not supported.

**Table 6:** Path coefficient of the research model.

**Hypothesis**	**Path hypothesis**			**Path coefficient**	**Path hypothesis**	**S.E**.	**C.R**.	***P*** **-value**	**Test results**
H1	IN	←	BPNS	−0.167	−0.201	0.038	−5.805	^***^	Supported
H2	AT	←	BPNS	−0.071	−0.054	0.044	−1.287	0.301	Not Supported
H3	SN	←	BPNS	−0.186	−0.216	0.065	−3.471	0.008	Supported
H4	PBC	←	BPNS	−0.079	−0.097	0.070	−1.397	0.210	Not Supported
H5	IN	←	AT	0.354	0.609	0.068	9.087	^***^	Supported

*** *P* < 0.001. BPNS, basic psychological needs satisfaction; AT, attitude to 4P; SN, subjective norm; PBC, perceived behavioral control; IN, intention to live in nursing homes.

Attitude has a significant positive effect on intention (β = 0.354, *p* < 0.001), the subjective norm has a significant positive effect on intention (β = 0.463, *p* < 0.001), and perceived behavioral control has a significant positive effect on intention (β = 0.295, *p* < 0.001), so H5 is supported.

### Mediating effect analysis

The mediating effect is not supported if the path from the independent variable to the mediating variable is insignificant. The results obtained from 4.3, H2 and H4 are not supported. So, the mediating effects of attitude and perceived behavioral control are not supported. H6 is not supported.

The bootstrap method is one of the most suitable methods for verifying the mediation effect (44, 45). Therefore, the bootstrapping method was used to verify the mediating effect. When the 95% confidence interval excludes 0, the effect is significant. Conversely, when the 95% confidence interval for the direct effect includes 0, the effect is insignificant. Under the premise that the total effect is significant, if both the indirect effect and the direct effect are significant, then the variable has a partial mediating effect; if the direct effect is not significant and the indirect effect is significant, then the variable has a full mediating effect (45).

Table 7 shows that the indirect effect value of BPNS_SN_IN is −0.086. The 95% confidence interval is (−0.156, −0.011) and (−0.159, −0.012), excluding 0, indicating that the indirect effect is significant, so H7 is supported. The direct effect of BPNS_IN is −0.171. After adding the subjective norm, the total effect of BPNAU_IN is −0.318, so the Subjective norm has a partial mediating effect.

**Table 7:** Bootstrap results of mediating effect.

**Path**	**Standardized effect value**	**Bias-Corrected 95%CI**		**Percentile 95%CI**	
		**Lower bounds**	**Upper bounds**	**Lower bounds**	**Upper bounds**
Total Effect					
BPNS_IN	−0.318	−0.472	−0.139	−0.481	−0.143
Indirect Effect					
BPNS_AT_IN	−0.027	−0.091	0.031	−0.093	0.033
BPNS_SN_IN	−0.086	−0.156	−0.011	−0.159	−0.012
BPNS_PBC_IN	−0.024	−0.070	0.023	−0.073	0.024
Direct Effect					
BPNS_IN	−0.171	−0.273	−0.063	−0.275	−0.064

BPNS, basic psychological needs satisfaction; AT, attitude to 4P; SN, subjective norm; PBC, perceived behavioral control; IN, intention to live in nursing homes.

## Discussions and conclusion

It is worth mentioning that our research found that basic psychological needs satisfaction harms the Chinese older adults' intention to live in nursing homes. This finding is consistent with the hypothesis of this study. Meeting people's basic psychological needs can promote their physical and psychological wellbeing (17). In the whole life cycle of older adults, “living in a nursing home” manifests regression and weakening of personal ability. Therefore, the higher the basic psychological needs satisfaction of the older adults, the less willing they are to live in nursing homes.

The surprising correlation is basic psychological needs satisfaction did not affect the older adults' attitude to nursing homes. These results differ from previous studies (12, 13). They may somewhat limit these findings. It could be argued that the positive results were due to the attitude scale using the attitude's 4Ps scale (attitude to nursing homes' product, price, place and promotion). The scale includes 27 items with four dimensions: prices, product, place and promotion. Compared with the attitude scale in TPB (21, 46), this scale has a different focus (on the marketing 4Ps) and is more detailed. These differences mean that study findings need to be interpreted cautiously. Therefore, this result may be explained by the fact that when people's attitude toward a thing is measured from the perspective of marketing 4Ps, people's attitudes may not be related to the degree of satisfaction of basic psychological needs but only related to the 4Ps of the thing itself.

In addition, basic psychological needs satisfaction does not affect perceived behavioral control, which does not support previous studies (25, 47). Reviewing the definition of perceived behavioral control, it stands for perceptions of whether the behavior is “difficult” and under its own control (21). They are combined with the requirements of staying in a Chinese nursing home. There may be several aspects of the perceived behavioral control of older adults over admission to nursing homes. First, whether their pension is sufficient to cover the cost of living in suitable nursing homes and daily living. An ethnographic study of China's older adults' care institutions addressed older adults' perceptions of their financial and caregiving choices. Older adults expressed that soaring medical costs determine their caregiving choices, “if I use my pension to pay for a nursing home, I cannot afford medicine” (48).

Similarly, if older adults have insufficient or no pensions, their perceived behavioral control may be the extent to which their family members (i.e., adult children) are willing to pay for nursing home expenses. A study of institutional care for older adults from rural China showed that the cost of entering a nursing home for rural older adults is mostly paid by their adult children (49). Second, the extent to which older adults' family members are willing to care for them at home is also one factor that influences the perceived behavioral control of Chinese older adults. In the Confucian culture, older Chinese adults are typically cared for by family members (50). But because of the “one-child policy” and the rise of “female professionals,” family members have a heavier burden of caring for the older adults at home than before (51). Studies have shown that when the caregivers are adult children rather than spouses or “other related” there is a small risk of placement in a nursing home (52). Third, whether there are any restrictions on admission to nursing homes may also be related to the perceived behavioral control of older adults. For example, care needs levels, and funding review. Research from rural China shows that most nursing homes refuse to admit frail and demented older adults because of a lack of skilled nursing staff (53). Nevertheless, not all nursing homes do not admit frail and demented older adults. There is no universal set of standards for admission to nursing homes, and each nursing home has different admission standards and corresponding charging standards. There is no charge for admission to public nursing homes in China, so the older adults need to meet the requirements of being over 60 years old and having no infectious diseases, mental illnesses, etc., and the older adults need to apply for admission in advance. Private nursing homes need to be charged, and admission generally requires the issuance of a medical examination report from the hospital and the payment of a deposit. Private nursing homes in some cities need to apply in advance and wait due to scarcity and good service. Fourth, the perceived behavioral control of older adults was also related to how long the waiting list for admission to a nursing home is. Some private nursing homes have long waiting lists, and the final list may be skewed toward the more successful (54).

An explanation for the uncorrelated relationship between basic psychological needs satisfaction and perceived behavioral control is that older adults may simply have no idea whether admission to a nursing home is “difficult” for them. A Chinese study shows that placing an older adults in a nursing home is often a deliberate decision made by adult children (55). Therefore, we found that the perceived behavioral control of older adults' nursing home admission contains many factors.

Subjective norm mediates basic psychological needs satisfaction and intention to live in nursing homes in our topic. That is, the higher the satisfaction of the basic psychological needs of the older adults, the less pressure they perceive “you should live in a nursing home” from significant others around them, and the lower their intention to live in a nursing home. Conversely, the lower the satisfaction of the basic psychological needs of the older adults, the higher the pressure they perceive “you should be admitted to a nursing home” from significant others around them, and the higher their intention to live in nursing homes. This is an unexpected outcome: the subjective norm is more potent than we thought. The Chinese collectivist culture may explain this result that the subjective normative effect of collectivism is stronger than individualism (56).

## Research implications

### Theoretical implications

The findings from this study make several contributions to the current literature. First, there is no research integrating motivation sequences to explore the older adults' intention to live in nursing homes. This research fills this gap and enriches the BPNT-TPB model's usage context. Second, our research found that there may be no correlation between basic psychological needs satisfaction and attitudes. Although this may be related to our choice of the marketing four Ps scale for attitudes rather than the traditional attitude scales in TPB, it is still an unexpected finding that gives us theoretical implications. Last, our findings also highlight the importance of subjective norms in research in the context of China's culture, where collectivism is stronger than individualism. These findings will provide theoretical suggestions for future related research in China.

### Practical implications

This research should be precious to the specific government departments and entrepreneurs who wish to attract residents. On the one hand, the lower level of the basic psychological needs satisfaction of the older adults, the higher intention they admit to nursing homes. So, entrepreneurs need to find ways to attract older adults who do not meet their basic psychological needs by building an environment that satisfies autonomy, relatedness, and competence in nursing homes. Marketers should try to segment older adult populations and develop appropriate marketing plans for older adults who do not meet basic psychological needs in their daily lives.

On the other hand, because of the only mediating effect of subjective norm, our study suggests that the opinions of significant others are also crucial for older adults' occupancy. So, entrepreneurs and marketers should identify significant others around older adults, their adult children, spouses, and siblings. Moreover, develop service plans and marketing programs that meet the needs of significant others to increase occupancy. For example, the perception that “sending parents to nursing homes is unfilial” still exists in Chinese society (57). So, if marketers ramped up the promotion that sending older adults to nursing homes for more professional services is more filial, it could increase occupancy rates for the whole industry. Besides, some adult children will experience a series of psychological torture after sending older adults to nursing homes, and grief and guilt are common manifestations (58). Furthermore, research shows that older adults experience a series of psychological reactions when they move to a nursing home, “fear, struggle, compromise, acceptance, and contribution” (59). These bad experiences are pain points that need to be addressed urgently. If entrepreneurs install a remote video system in nursing homes, significant others can video chat with older adults to ease the sense of loss of sudden separation. Significant others can also continue to express their concern for the older adults by video chatting. This can help significant others, and older adults go through the difficult relocation period smoothly. Besides, this may increase the satisfaction of the significant others and the older adults in the nursing home. Thereby enhancing the individual competitiveness of nursing homes.

## Study limitations and future research

Three limitations are worth highlighting. First, this study uses a cross-sectional survey to test the hypotheses. The researcher needs to be aware of possible time-sensitive relationships between the variables. Second, the sample of this study is the older adults in Henan Province, China. There may be regional bias in the findings. If more general findings are needed, other regions need to be studied. Previous studies suggested that culture affects people's perceptions, thoughts and behaviors (60, 61). This can provide a theoretical basis for studying the different behaviors due to different cultures and norms that affect the older adults' needs and perceptions of nursing homes. Third, the research instruments used in this study may have limitations. The attitude's 4Ps scale was used to measure the attitude of the older adults toward nursing homes, and the results showed that the attitude of the older adults toward nursing home marketing 4Ps was not affected by the degree of satisfaction of basic psychological needs. However, this result does not equate to the fact that the older adults' attitude toward nursing homes is not affected by the degree of satisfaction with basic psychological needs. Future studies can change the attitude scale for research if more objective results are needed. If future research requires more objective results, the attitude scale can be changed for research.

In addition, this study also found the only partial mediating role of subjective norms (i.e., opinions and pressures from significant others) in the relationship between basic psychological needs satisfaction and intentions to live in nursing homes. This result suggests that significant others are important for older adults to have specific intentions. Consequently, future research can further investigate the factors that predict the attitudes of the older adults' “significant others.” Besides, some predictors that we did not include in the study may also be helpful for research in this direction. Distance from nursing homes to their own homes may be a good predictor of essential psychological needs satisfaction. Future research can consider integrating distance into the basic psychological needs model. Finally, future studies could translate live intentions into behaviors through longitudinal study designs.

## Data availability statement

The original contributions presented in the study are included in the article/supplementary material, further inquiries can be directed to the corresponding author.

## Ethics statement

The studies involving human participants were reviewed and approved by the Rajamangala University of Technology Rattanakosin. The patients/participants provided their written informed consent to participate in this study.

## Author contributions

ML conceived the research, collected the data, performed the data analysis and interpretation, wrote, and revised the manuscript. JD guided the research ideas, helped revise the manuscript, and intensively edited the language of the manuscript. RL helped revise the manuscript and guided the journal selection. NW contributed to the development of research ideas. All authors contributed to the article and approved the submitted version.

## Conflict of interest

The authors declare that the research was conducted in the absence of any commercial or financial relationships that could be construed as a potential conflict of interest.

## Publisher's note

All claims expressed in this article are solely those of the authors and do not necessarily represent those of their affiliated organizations, or those of the publisher, the editors and the reviewers. Any product that may be evaluated in this article, or claim that may be made by its manufacturer, is not guaranteed or endorsed by the publisher.
